# Better prediction of functional effects for sequence variants

**DOI:** 10.1186/1471-2164-16-S8-S1

**Published:** 2015-06-18

**Authors:** Maximilian Hecht, Yana Bromberg, Burkhard Rost

**Affiliations:** 1 Department of Bioinformatics & Computational Biology, Technische Universität München, Boltzmannstr. 3, 85748 Garching/Munich, Germany; 2 Department of Biochemistry and Microbiology, Rutgers University, 76 Lipman Dr, New Brunswick, NJ 08901, USA; 3 Department of Genetics, Rutgers University, 145 Bevier Road, Piscataway, NJ 08854-8082, USA; 4 Institute of Advanced Study (TUM-IAS), Lichtenbergstr. 2a, 85748 Garching/Munich, Germany & WZW - Weihenstephan, Alte Akademie 8, Freising, Germany

**Keywords:** functional effect prediction, variant effect, neural network, from sequence, SNP effect

## Abstract

Elucidating the effects of naturally occurring genetic variation is one of the major challenges for personalized health and personalized medicine. Here, we introduce SNAP2, a novel neural network based classifier that improves over the state-of-the-art in distinguishing between effect and neutral variants. Our method's improved performance results from screening many potentially relevant protein features and from refining our development data sets. Cross-validated on >100k experimentally annotated variants, SNAP2 significantly outperformed other methods, attaining a two-state accuracy (effect/neutral) of 83%. SNAP2 also outperformed combinations of other methods. Performance increased for human variants but much more so for other organisms. Our method's carefully calibrated reliability index informs selection of variants for experimental follow up, with the most strongly predicted half of all effect variants predicted at over 96% accuracy. As expected, the evolutionary information from automatically generated multiple sequence alignments gave the strongest signal for the prediction. However, we also optimized our new method to perform surprisingly well even without alignments. This feature reduces prediction runtime by over two orders of magnitude, enables cross-genome comparisons, and renders our new method as the best solution for the 10-20% of sequence orphans. SNAP2 is available at: https://rostlab.org/services/snap2web

**Definitions used:**

Delta, input feature that results from computing the difference feature scores for native amino acid and feature scores for variant amino acid; nsSNP, non-synoymous SNP; PMD, Protein Mutant Database; SNAP, Screening for non-acceptable polymorphisms; SNP, single nucleotide polymorphism; variant, any amino acid changing sequence variant.

## Introduction

Some sequence variations matter, changing native protein function or disease-causing potential, while others do not [1]. The distinction between the variants that change protein function and those that are neutral is one key to making sense of the deluge Next Generation Sequencing (NGS) or Deep Sequencing data. Many methods have been developed that address this challenge, spanning a wide range of goals and applications. Some tools are focused on non-coding regions [2-4]; others focus on coding regions and predict the effects of single amino acid variants (non-synonymous single-nucleotide polymorphisms, nsSNPs, or single amino acid substitutions, SAAS) on aspects such as protein structure [5], stability [6-8], binding affinity [9], and function [10,11]. Some methods focus exclusively on the human genome [12,13] and some aspire to identify disease-causing variants [14-16]. Applications to personalized health are obviously important considerations for the developers of such tools. Generally, today's methods are able to distinguish between a set with 100 disease-causing and another with 100 less impacting variants [17,18]. However, identifying one or several variants in an individual responsible for a certain disease is often beyond our reach. Methods have improved significantly by using more protein and variant annotations, as demonstrated in particular in the advance from PolyPhen [12] to PolyPhen-2 [13]. Despite many advances, good data remains missing, in particular careful annotations of variant neutrality, partially because it is to difficult to carry out "negative experiments" (absence of change [19]).

The best variant effect prediction methods typically use evolutionary information, and a wide variety of features descriptive of protein function and structure. Performance decreases substantially for proteins without informative multiple alignments. Today few human proteins do not map to well-studied sequence families. However, most fully sequenced organisms, predominantly prokaryotic, contribute a substantial fraction of "orphans" (10-20%) [20].

Today's state-of-the-art prediction methods focus on discerning disease-causing variants from the background variation. They, *e.g*. differentiate between human cancer-causing mutations and common variation. This implicitly disregards many variants with functional effects that are not associated with disease. In contrast, the current version of our SNAP (Screening for Non-Acceptable Polymorphisms) method, SNAP2, does not predict the variant effect as "disease or not" but rather as "change of molecular function or not". Similar to most experimental assays, SNAP2 does not directly connect "molecular change" to "impact on organism"; *i.e*. the goal is not to support statements of the type "this single variant improves survival rate". Also similar to many experimental methods, we avoid distinguishing gain-of-function from loss-of-function variants, as these outcomes are often subjective. For instance, gaining in the ΔΔG of binding does NOT imply a "better molecular function" and even the gain of "molecular function" might decrease survival. Here, we introduced several concepts each of which importantly improved over our previous method, SNAP [11]. SNAP2 outperforms its predecessor in three major aspects: better performance, better predictions without alignments, and many orders of magnitude lower runtime.

## Methods

### Data sets

The training set for SNAP2 resembled that used for development of the original SNAP [11]. In particular, we used the following mixture: variants from PMD (the Protein Mutant Database [21]), residues differing between enzymes with the same experimentally annotated function according to the enzyme classification commission (EC), retrieved from SWISS-PROT [22,23], variants associated with disease as annotated in OMIM (Online Mendelian Inheritance in Men [24]), and HumVar [25].

*PMD*. We extracted all amino acid changing variants from the Protein Mutant Database [21] (PMD) and mapped these to their corresponding sequences. PMD annotations with 'no change' ('=') qualification (function equivalent to wild-type) were assigned to the 'neutral' class, while variants with any level of increase ('+', '++', '+++') or decrease ('-', '- -', '- - -') in function were assigned to the 'effect' class. Variants with conflicting functional effect annotations were also classified as 'effect'. This approach identified 51,817 variants (neutral: 13,638, effect: 38,179) in 4,061 proteins.

*EC*. 74% of the PMD data were 'effect' annotations. We balanced this with evidence for neutral variants from enzyme alignments. Assume independent experiments reveal two enzymes to have the same function, i.e. the same EC number (Enzyme Commission number [26]). If these two proteins are very sequence similar, most variants between them are likely 'neutral' with respect to the EC number. While not always correct, the procedure creates a set heavily enriched in truly 'neutral' variants. To turn this concept into data, we aligned all enzymes with experimentally assigned EC numbers in SWISS-PROT [22] using pairwise BLAST [27]. We retrieved all enzyme pairs with pairwise sequence identity >40% and HSSP-values>0 [28-30]. This yielded 26,840 'neutral' variants in 2,146 proteins [11].

*Disease*. We extracted 22,858 human disease-associated variants in 3,537 proteins from OMIM [24] and HumVar [25]. All disease-associated variants were classified as 'effect'. For many of these variants the change in protein function has not explicitly been demonstrated. These variants may be not causative but, possibly, in linkage disequilibrium with the actual disease-causing variants. Alternatively, they may be affecting splice-sites and/or regulatory elements in the DNA, finally showing up as amino acid substitutions. Hence, by compiling these into the effect class we may be over-estimating functional changes. However, we previously established that relationships to disease provide much stronger evidence for functional effect of variants than any other experimental evidence [17]. Thus, disease variants are clearly strongly enriched in functional significance.

*Protein specific studies*. We also included data from comprehensive studies of particular proteins, namely LacI repressor from *Escherichia coli *[31] (4,041 variants) and the HIV-1 protease [32] (336 variants). Variants functionally equivalent to wild-type were considered 'neutral'; all others were deemed 'effect'. These variants were not included in training, overlaps (same variant in one of the sets above and these) were removed.

*Evaluation sets*. We created three subsets of our data for evaluation/development of SNAP2. First, *PMD *+ *EC + Disease *were compiled into one comprehensive set termed *ALL *with 101,515 variants (40,478 neutral, 61,037 effect) in 9,744 proteins. We also split the *PMD *data into two subsets: one containing only human mutations (*PMD_HUMAN; *9,657 variants in 678 human sequences) and one consisting of all others (*PMD_NONHUMAN*; 42,160 variants in 3,383 sequences).

### Cross-validation

We clustered our data such that the sets used for training (optimizing neural network connections), cross-training (picking best method) [33,34], and testing (results reported) were not significantly sequence similar. Toward this end, we all-against-all PSI-BLASTed all proteins in our data sets and recorded all hits with E-values<10^-3^. Starting with these, we built an undirected graph, where vertices are proteins and edges link vertices to the corresponding BLAST hits. We then clustered all proteins using single linkage clustering; *i.e*. all connected vertices were assigned to the same cluster. This yielded 1,241 clusters of related protein sequences with 1 to 1,941 members. We randomly grouped the clusters into ten subsets of roughly similar size. This approach ascertained that no two proteins between any sets were significantly sequence similarity. Due to extremely varied cluster sizes one of these subsets was nearly three times larger than the others. This imbalance was acceptable since the cross-validation procedure ensured sufficiently more training data than testing data in each rotation. In tenfold cross-validation, we rotated through the subsets using eight for training, one for cross-training and the tenth for testing, such that each subset (and therefore each protein) was used for testing exactly once. As a result no variant, protein sequence, or even close homologue, was ever used simultaneously for training and testing. All performance estimates that we reported were solely based on the testing set.

### Prediction method

We applied the different machine learning tools in the WEKA suite [35] to our data with default parameters. Support Vector Machines (SVMs) and Neural Networks performed similarly and slightly better than Decision Trees and Random Forests. Due to runtime efficiency, we decided to proceed with standard neural networks. As in similar applications [11,36], we used two output units: one for 'neutral', the other for 'effect'. All free network parameters were optimized on the training (optimizing connection weights) and cross-training (optimizing number of hidden units, learning rate, and momentum; stop training before over-fitting) sets. Tenfold cross-validation implies training ten networks: which one to use for future applications? Taking the "best" of the ten risks over-training. We avoid this by using all ten networks to predict for new proteins, compiling separate averages for 'neutral' and 'effect' over all ten networks. The final prediction is the difference between these averages that ranges from -100 (strongly predicted 'neutral') to +100 (strongly predicted 'effect').

### Input features

Biophysical amino acid features and predicted aspects of protein function and structure help to predict the impact of variants. Not knowing connections between residues (our method does not require the knowledge of 3D structures), we scanned sliding windows of up to 21 consecutive residues around the central variant position. We compiled the original SNAP features: biophysical amino acid properties, explicit sequence, PSIC profiles [37], secondary structure and solvent accessibility [38-40], residue flexibility [41], and SWISS-PROT annotations. Additionally, we introduced new features for SNAP2: amino acid properties as provided by the AAindex database [42], predicted binding residues [43], predicted disordered regions [44], proximity to N- and C-terminus, statistical contact potentials [45], co-evolving positions, residue annotations from Pfam [20] and PROSITE [46], low-complexity regions, and other global features such as secondary structure and solvent accessibility composition (Additional File 1, *Input feature calculation*).

### Feature selection

In order to determine the optimal feature combination, we systematically sieved through our feature space using greedy bottom-up feature selection. For the following procedure one of the ten training folds (specific to each network) was kept out so that it had no part in feature selection and parameter optimization at any point. We trained ten networks, using 9 of the 10 data subsets: 8 for training and 1 for cross-testing as described above, using each feature and selecting the highest scoring feature separately for each network (highest AUC, Area Under ROC Curve, in cross-training). In the next round, the selected feature was combined with each of the remaining features to train another round of ten networks and the best performing combination of features was selected - again, for each network separately. We repeated until no additional feature improved performance. We considered different sequence window sizes for each feature independently; *i.e*. each feature could be selected in a window of w = 1,5,9,13,17, or 21 consecutive residues around the observed variant at the center of the window.

We tried to avoid local maxima in training via the following steps: S1: Train with balanced data sets [38,40]. S2: Determine the AUC on the cross-training set after each repetition. Record the step with maximal AUC. S3: Train and determine AUC for the cross-training set at least another ten repetitions from the highest-scoring step. Repeat S2-S3 until no additional improvement is recorded.

We collected all features that improved performance on any of the individual networks into a single combined feature set and trained all networks on this set. In a subsequent backward elimination, we removed all features the removal of which did not alter the average overall prediction accuracy. After determining the final feature space, we optimized the number of hidden nodes, learning rate, and learning momentum to obtain the best-performing network architecture. As an exhaustive screening of the entire parameter space was not intended, we heuristically selected parameter combinations for optimization: learning rate 0.005-0.1, learning momentum 0.01-0.3, and hidden nodes 10-100. The best-performing architecture for each network, as determined by its performance on the corresponding cross-training set, was chosen for the final method.

Finally, we tested the resulting trained networks (of specific feature space and the network architecture each) against the test sets that were initially kept out of feature selection and parameter optimization. Since the performance on these test sets did not differ significantly from that estimated during the optimization procedure, we concluded that we had not over-fitted the networks to the data.

### Predicting effects without alignments

We repeated the above feature selection restricted to global features (features based on the entire protein, such as amino acid and secondary structure compositions), amino acid indices, alignment-free secondary structure predictions, and the biophysical amino acid properties. We explicitly left out evolutionary information. We wanted to add a generic average for 'potential effect'. Toward this end, we used the complete version of SNAP2 to predict effects for all possible variants at each residue position in our entire *ALL *set. From these results, we generated a novel amino acid substitution matrix of effect probabilities [47] which we included as an additional feature in the feature selection. This procedure was aimed at developing a method that can be applied without alignments. The resulting method (SNAP2_noali_) predicts functional effects using only single sequences. Note that our SNAP2 implementation selects the best method given the available information, SNAP2 by default and SNAP2_noali _for orphans. In the latter case, users are notified about the possibly reduced accuracy of predictions.

### Performance measures

We evaluated performance via a variety of measures. For simplicity, we used the following standard annotations: True positives (TP) were correctly predicted experimental 'effect' variants, while false positives (FP) were experimentally 'neutral' substitutions incorrectly predicted to have an effect. True negatives (TN) were correctly predicted neutrals and false negatives (FN) were effect variants incorrectly predicted to be neutral. Here, like everywhere else in computational biology, we accept incorrect estimates originating from the triviality that "not observed" does not always imply "not existing", *i.e*. some of the FP might have an effect that was not experimentally tested. We calculated accuracy (precision) and coverage (recall) separately for 'effect' (Eqn. 1) and 'neutral' (Eqn. 2) predictions:

(1) $$\begin{array}{c} Accuracy_{effect}=Precision_{effect}=Positive\;predictive\;value=\frac{TP}{TP+FP}\\ Coverage_{effect}=Recall_{effect}=Sensitivity=\frac{TP}{TP+FN} \end{array}$$

(2) $$\begin{array}{c} Accuracy_{neutral}=Precision_{neutral}=Negative\;predictive\;value=\frac{TN}{TN+FN}\\ Coverage_{neutral}=Recall_{neutral}=Specificity=\frac{TN}{TN+FP} \end{array}$$

We used the F-measure (F1-Score; Eqn. 3) to asses 'neutral' and 'effect' variants individually. Combined performance was measured by the overall two-state accuracy (Q2; Eqn. 4) and the Matthews Correlation Coefficient (MCC; Eqn. 5).

(3) $$\begin{array}{c} F_{effect}=2\;\cdot \;\frac{precision_{effect}\;\cdot \;recall_{effect}}{precision_{effect}\;+\;recall_{effect}}\\ F_{neutral}=2\;\cdot \;\frac{precision_{neutral}\;\cdot \;recall_{neutral}}{precision_{neutral}\;+\;recall_{neutral}} \end{array}$$

(4) $$Q_{2}=Accuracy=\frac{TP+TN}{\left(TP+FP+TN+FN\right)}$$

(5) $$MCC=\frac{TP\;\cdot \;TN\;-\;FP\;\cdot \;FN}{\sqrt{\left(TP+FP\right)\left(TP+FN\right)\left(TN+FP\right)\left(TN+FN\right)}}$$

Standard deviation and error for all measures were estimated over n = 1000 bootstrap sets; for each set we randomly selected 50% of all variants from the original test set without replacement. Note that due to over-representation of certain protein families, in our experience, bootstrapping without replacement typically yields error estimates that are more accurate than those with replacement. Standard deviation was calculated as the difference of each test set (x_i_) from the overall performance 〈*x*〉 (Eqn. 6). Standard error was calculated by dividing σ by the square root of sample size (Eqn. 7).

(6) $$\text{Standard deviation }\left(\text{SD}\right)=\sqrt{\frac{\sum {\left(x_{i}-\langle x\rangle \right)}^{2}}{n}}$$

(7) $$\text{Standard error }\left(\text{SE}\right)=\frac{SD}{\sqrt{\left(n-1\right)}}$$

The reliability index (RI; Eqn. 8) for each prediction was computed by normalizing the difference between the two output nodes (one for 'neutral', the other for 'effect') into integers between 0 (low reliability) and 10 (high reliability):

(8) $$RI=10\;\cdot \;|int\left(Output_{effect}-Output_{neutral}\right)|$$

## Results

### SNAP2 significantly improves predictions

First, we assessed the performance of SNAP2 via cross-validation on the original SNAP data. Here, we observed a performance increase over our original SNAP, originating from novel features used in SNAP2. However, by adding in more and better variant data, we found a further (and significantly higher) improvement in performance over SNAP. Many computational methods predict variant effects. As most of these methods focus on predicting disease-associated variants, assessing their performance on our data is inappropriate. Therefore, we explicitly compared SNAP2 only to widely used methods that explicitly aim at the prediction of functional effects: SIFT [10] and PolyPhen-2 [13]. All estimates for the performance of SNAP2 given in this work are based on full cross-validation testing, *i.e*. on data never used for any step in the development. Note that this is not true for other methods in our comparisons.

**Figure 1:**
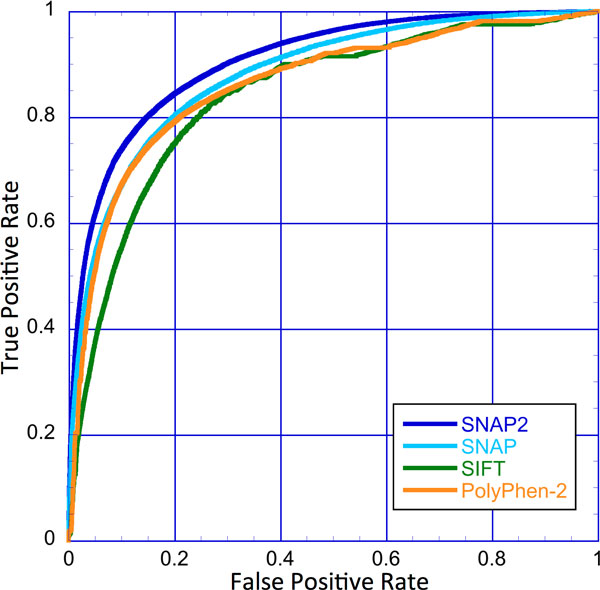
**SNAP2 performs best for the ALL data set**. This figure shows performance estimates for the *ALL *data set. Our new method SNAP2 (dark blue, AUC = 0.905) outperforms its predecessor SNAP (light blue, AUC = 0.880), PolyPhen-2 (orange, AUC = 0.853) and SIFT (green, AUC = 0.838) over the entire spectrum of the Receiver Operating Characteristic (ROC) curve. Curves are significantly different from each other at a significance level of P < 10-4 as measured by the DeLong method [59]. All SNAP2 results were computed on the test sets not used in training after a rigorous split into training, cross-training and testing. Results for PolyPhen-2 and our original SNAP included some of those proteins in their training, suggesting over-estimated performance.

On the ALL data set (Methods), SNAP2 outperformed its predecessor SNAP [11], as well as both PolyPhen-2 and SIFT (Figure 1). However, the direct comparison is complicated due to a variety of issues. Firstly, the original SNAP was trained on PMD, suggesting a performance overestimate. Secondly, SIFT scores were normalized and optimized for simple defaults. This is implicitly ignored by showing ROC-curves that provide values for a wide set of thresholds that had been deemed non-optimal by the developers. Thirdly, PolyPhen-2 is optimized on human variants that account for only 25% of our *ALL *data. For these, we over-estimate PolyPhen-2's performance. Although the authors assumed that PolyPhen-2 would perform similarly for other eukaryotes, it might not. To address these complications we compared the methods using additional data sets.

### Performance differed between the human and non-human PMD data

The F-measure for predicting effect (F_effect_, Eqn. 3), the two state-accuracy (Q2, Eqn. 4), and the Matthew's correlation coefficient (MCC, Eqn. 5) were slightly higher for SNAP2 when tested on the non-human than on the human set (Table 1). For the human PMD data, PolyPhen-2 performed on par with SNAP2, while SIFT was best for predicting neutrals. For the non-human data, SNAP2 was either on par (F_neutral_, Eqn. 3) or outperformed (F_effect_, Q2, MCC) all other methods (Table 1). Again, this comparison is not entirely fair to SNAP2 and SIFT since the human PMD variants overlapped substantially with the PolyPhen-2 training set, i.e. Table 1 likely over-estimates PolyPhen-2.

**Table 1:** Method performance on PMD *

	*Method*	*F_effect _(Eqn. 3)*	*F_neutral _(Eqn. 3)*	*Q2 (Eqn. 4)*	*MCC (Eqn. 5)*
human	*SNAP2*	**78.0% ± 0.6**	46.3% ± 1.3	**68.8% ± 0.7**	0.24 ± 0.01
	
	*PolyPhen-2*	78.4% ± 0.4 **	45.1% ± 1.1 **	68.9% ± 0.5 **	0.23 ± 0.01 **
	
	*SNAP*	74.9% ± 0.5	46.7% ± 1.1	65.8% ± 0.6	0.22 ± 0.01
	
	*SIFT*	72.2% ± 0.6	**49.0% ± 1.0**	63.6% ± 0.6	0.23 ± 0.01

non-human	*SNAP2*	**79.9% ± 0.3**	45.8% ± 0.8	**70.7% ± 0.4**	**0.26 ± 0.01**
	
	*PolyPhen-2*	77.1% ± 0.4	44.7% ± 0.8	67.6% ± 0.5	0.22 ± 0.01
	
	*SNAP*	77.2% ± 0.3	45.5% ± 0.9	67.9% ± 0.5	0.23 ± 0.01
	
	*SIFT*	77.0% ± 0.3	45.8% ± 0.8	67.7% ± 0.4	0.23 ± 0.01

* Data set consisting of 9,657 variants (2,788 neutral, 6,869 effect) from 678 human proteins in the top rows and 42,160 variants (10,850 neutral, 31,310 effect) from 3,383 non-human proteins in the bottom rows. For each measure and species group, significantly best results are highlighted in bold. Measures with no bold highlighting indicate absence of a statistically significant best performer.

** Values might over-estimate performance for PolyPhen-2 due to overlap between data set used here and one used for training PolyPhen-2.

### Blind method combinations might be worse than a good single method

**Figure 2:**
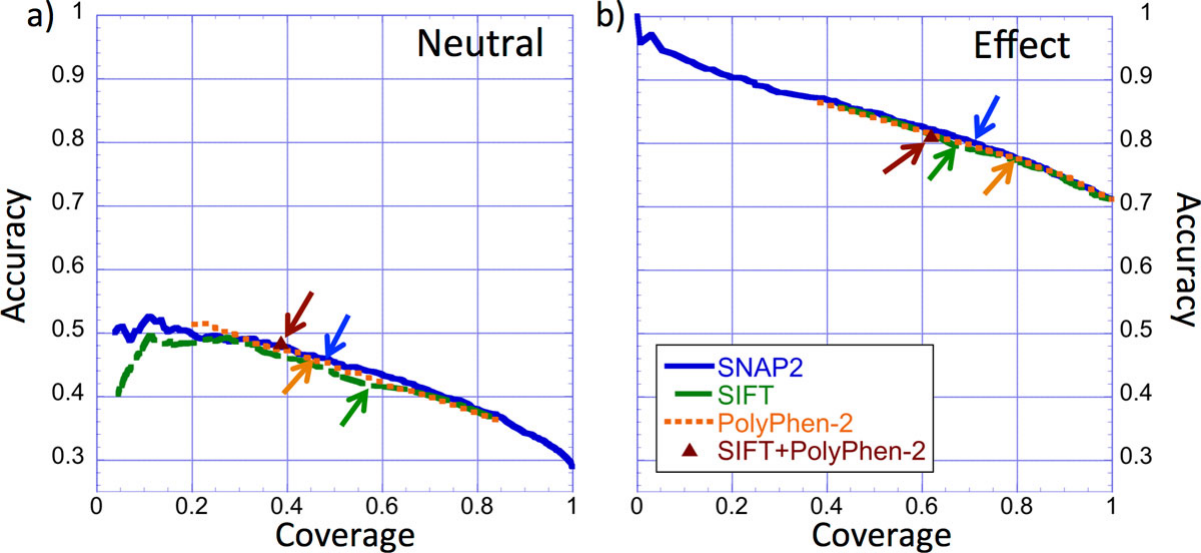
**Naïve combination is not better than individual methods for *PMD_HUMAN *data**. This figure shows accuracy-coverage curves for the PMD_HUMAN data. The x-axes indicate coverage (also referred to as 'recall'; Eqn. 1.2), *i.e*. the percentage of observed neutral (a) and of observed effect (b) variants that are correctly predicted at the given threshold. The y-axes indicate accuracy (also referred to as 'precision'; Eqn. 1.2), *i.e*. the percentage of neutral (a) and effect (b) variants among all variants predicted in either class at the given threshold. Arrows mark the performance at the default thresholds for our new method SNAP2 (dark blue), for SIFT (green), and for PolyPhen-2 (orange). A brown triangle/arrow marks the performance of a (non-optimized) method that combines PolyPhen-2 and SIFT. This combination did not perform better than SNAP2 alone (brown triangle vs. blue SNAP2 curves).

If in doubt which method is best, users often mix several methods. One strategy is to exclusively consider predictions for which several methods agree. We assessed the benefit of this strategy by applying SNAP2, SIFT and PolyPhen-2 on the *PMD_HUMAN *data set. All methods performed significantly worse for neutral than for effect variants. This can largely be attributed to the difference in the number of variants. The combination of SIFT and PolyPhen-2 improved slightly over SIFT alone for neutral variants (green curve vs. brown arrow/triangle in Figure 2A) and, in terms of accuracy (Eqn. 2) over PolyPhen-2 alone (orange curve vs. brown arrow/triangle in Figure 2A). However, for effect variants combining PolyPhen-2 and SIFT did not improve over the individual methods at all. Moreover, throughout the curves (Figure 2) of both neutral and effect variants, the combined method did not improve over using SNAP2 alone. Methods such as PredictSNP [48], Condel [49], and MetaSNP [50] have been explicitly optimized to combine different methods, mostly to annotate disease-variant relationships (as opposed to functional changes). Such *meta-*methods often tend to improve over the simple combinations individually attempted by many users and tested here.

### SNAP2 is clearly best for difficult cases

Although overall performance levels were similar for all methods tested on the *ALL *data set, the actual predictions for a single variant differed substantially between methods. Variants for which methods agree could be considered "easy" (every method right) or "unsolvable" (no method right). In contrast, variants for which methods disagree could be considered "difficult". This classification yielded 67,912 ***easy ***(~68% of the total; 27,370 neutral and 40,542 effect), 9,624 ***unsolvable ***(~10% of the total; 4,750 neutral and 4,874 effect), and 22,625 ***difficult ***variants (~22% of the total; 7,504 neutral and 15,121 effect). SNAP2 outperformed others on the difficult cases, correctly predicting 69%, as compared to SNAP with 53% and SIFT with 41% compared to 53±1% for random.

**Figure 3:**
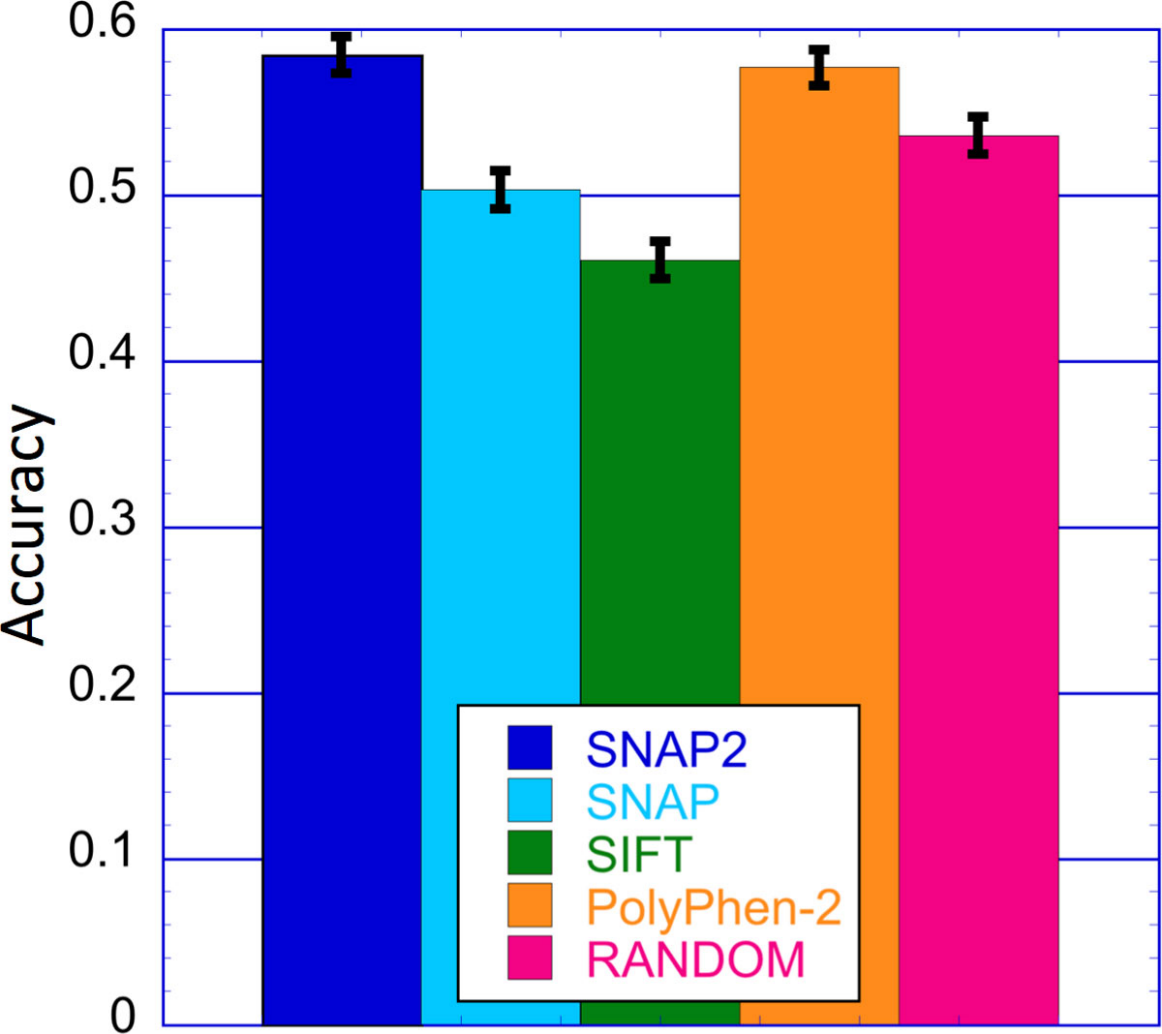
**SNAP2 and PolyPhen-2 are best for difficult human variants**. Bars mark the two-state accuracy (Q2; Eqn. 4) at the default thresholds for SNAP2 (dark blue), SNAP (light blue), SIFT (green), and PolyPhen-2 (orange). Random prediction performance assuming 60:40 effect:neutral background are given in pink. Analysis is based on 3,963 'difficult' cases (2,589 effect; 1,374 neutral) from *PMD_HUMAN *set. Difficult cases were defined as variants where any of the above method's predictions disagreed; *i.e*. cases where not all methods, excluding random, gave the same prediction.

We repeated the same analysis for the *PMD_HUMAN *subset (Figure 3). For the 3,963 human variants (1,374 neutral and 2,589 effect) for which any two of the methods disagreed, SNAP2 and PolyPhen-2 were correct in ~58% of the cases compared to 50% for SNAP, 46% for SIFT and 44±1% for random predictions. Again, the PolyPhen-2 training set overlapped with these data, suggesting a performance over-estimate.

In this set of 3,963 human variants, 305 (45 neutral and 260 effect) were only correctly predicted by SNAP2. We investigated these cases in detail, and found that the effect variants in this set often localized to positions at which the variant residue had been observed in another protein in the alignment. For most methods, this implies "neutral" prediction. Indeed, SNAP2noali, the version of our method that does not use alignments, predicted 75% of these effect variants at over 90% accuracy, *i.e*. reached a performance substantially above its average for these cases. Thus, one important source of SNAP2 improvement for difficult cases originates from its use on various pieces of information, not just alignments. One example of this improvement is the R109Q variant in the IL4 sequence (interleukin-4 isoform 1 precursor; NCBI reference sequence: NP_000580.1), a pleiotropic cytokine produced by activated T-cells and involved in B-cell activation as well as co-stimulation of DNA synthesis [51]. Variations in this gene were shown to be associated with susceptibility to ischemic stroke [52] and knee osteoarthritis [53]. While our R109Q was not explicitly found to increase disease susceptibility, there is evidence [54] that it reduces T-cell proliferation and receptor binding activity. In this case, the variant glutamine is more conserved in the protein alignment than the human native arginine (11% Q vs. 8% R), making predictions difficult for methods that over-rely on alignments.

Another potential source of improvement, although one for which we could not find explicit and experimentally verified examples in our data, lies in the usage of information about co-evolving residues (Additional File 1, Input Feature Calculation). Specifically, some of the variant positions in this set exhibited (computationally-determined) strong correlations with other positions in the protein, suggesting that this particular feature also made a difference.

### Evolutionary information most important, other features vary

The input features related to evolutionary information were consistently most informative for SNAP2 (Additional File 1, Fig. SOM_1: SNAP2 vs. SNAP2_noali_). Which other input features best distinguished neutral from effect depended on the data set. This dependency might originate from annotation inconsistencies and/or set size differences or it might genuinely reflect the data. By selecting the best features separately for subsets of related proteins, we tried to differentiate between these alternatives. The majority of our subsets considered structural features (secondary structure and solvent accessibility) informative, followed by biophysical amino acid properties (more precisely: charge and hydrophobicity). However, the optimal window sizes (number of consecutive residues used as input) for these features differed. For instance, residue flexibility was considered informative by most subsets, but the optimal window size for this feature varied between three and nine residues around the variant.

The final SNAP2 network included the following features: global features (amino acid composition, secondary structure and solvent accessibility composition, and protein length), PSI-BLAST [27] profiles and deltas, PSIC [12] profiles and deltas (differences between mutant and wild-type residue annotations; see Methods for details), residue flexibility, sequence and variant profiles, disorder, secondary structure and relative solvent accessibility and their deltas, physicochemical properties (charge, hydrophobicity, volume, and their deltas), contact potential profiles and deltas, correlated positions and low complexity regions. In addition to these, SWISS-PROT [22] annotations and SIFT [10] predictions were included in SNAP2, if available. For the sequence-only network (SNAP2_noali_) the following features where included: amino acid composition, protein length, sequence and variant profiles, contact potential profiles and delta, volume and hydrophobicity along with the corresponding delta features as well as several amino acid indices from the AAindex [42] (Additional File 1, Table SOM_1).

### SNAP2_noali _important for many proteins

For eight proteins in the *ALL *data set we found fewer than five PSI-BLAST hits in UniProt when we first checked in Oct. 2012. On this tiny set SNAP2_noali _appeared better than SNAP2 (Eqn. 4: Q2_SNAP2noali _= 61% vs. Q2_SNAP2 _= 60%; Eqn. 5: MCC_SNAP2noali _= 0.19 vs. MCC_SNAP2 _= 0.17). PolyPhen-2 made predictions for only three of these eight proteins (103 variants, Q2_PolyPhen-2 _= 60%) and SIFT gave no predictions. Recently repeating the analysis, we found homologues for all eight. SNAP2, SIFT and PolyPhen-2 now outperformed SNAP2_noali_. Our "outdated" analysis was important. On the one hand, over 600 human proteins (~3% of all human) still find less than 5 homologues today. On the other hand, for most organisms for which we know the sequences, the corresponding value is much closer to 10-20%, i.e. millions of the proteins we know today can only be handled well by SNAP2_noali_.

For our entire training data, SNAP2_noali _reached Q2 = 68%, *i.e*. seven percentage points more than for the subset of proteins with small/no families (68% on ALL vs. 61% on NOALI eight protein set). About 10-20% of all proteins in newly sequenced organisms continue not to map anywhere else in today's databases [33,34,55]; for those 10-20% of proteins, SNAP2_noali _appears to be the best method available to predict the effect of mutations.

### Performance confirmed for additional data sets

We avoided over-optimistic performance estimates by removing sequence similarity between proteins used for method development (training/cross-training) and testing. In addition, we also tested our final method on two data sets of variants from the *Escherichia coli *LacI repressor and from the HIV-1 protease (Additional File 1, Table SOM_2). Given the small size and lack of diversity, these results are likely to be more error-prone than our cross-validation estimates. However, they provide independent evidence to estimate the performance of SNAP2: Q2 = 78% for 4,041 LacI variants and Q2 = 72% for 336 HIV-1 variants. None of these variants was used during method development. Moreover, our training data did not contain variants from any homologs of these proteins.

### Reliability index allows zooming into best predictions

**Figure 4:**
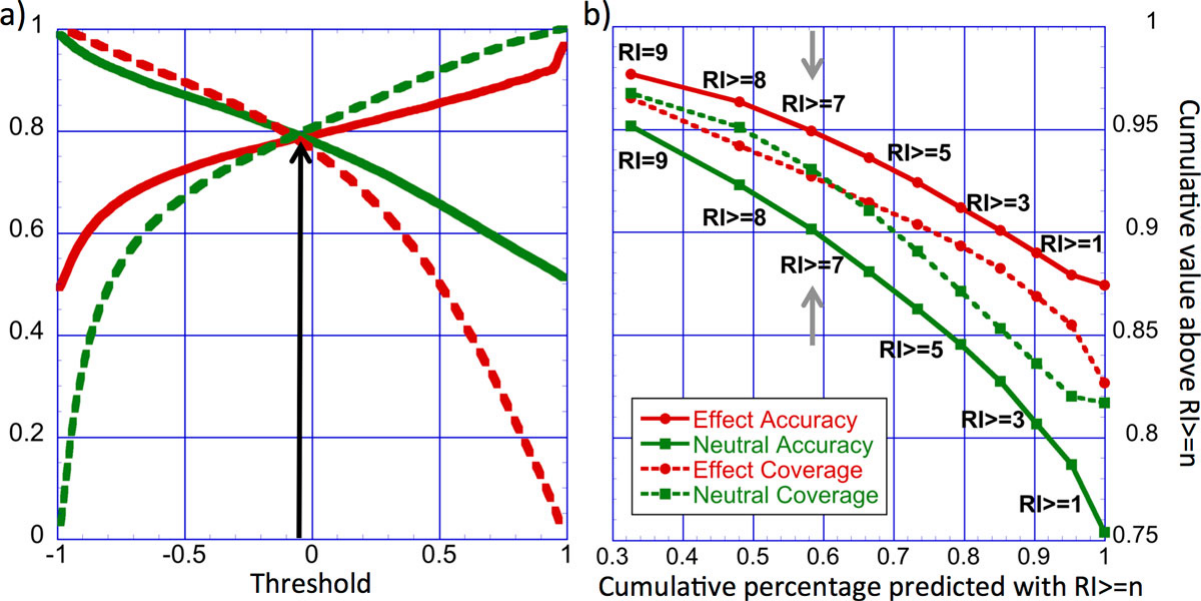
**SNAP2 threshold and reliability**. The reliability index provides a means of focusing on the most accurate predictions. Panel **(a) **shows SNAP2 performance on the balanced PMD/EC data set over the entire spectrum of accuracy (solid lines) and coverage (dotted lines) for both effect (red) and neutral (green) variants depending on the chosen threshold (x-axis). The default threshold was set to -0.05, where neutral and effect predictions performed alike (black arrow). By moving the decision threshold users can optimize predictive behavior towards their research needs: predictions at higher absolute scores (*e.g*. TP>0.5 or TN<-0.5) are much more likely correct but they are not available for all variants. Panel **(b) **directly relates the reliability index (RI) to the performance on our data. Shown is the cumulative percentage of predictions (x-axis) against accuracy (solid lines) and coverage (dotted lines) above a given reliability index (RI; Methods). Accuracy and coverage are shown separately for neutral (green) and effect (red) predictions. Each marker depicts a reliability threshold ranging from 0 (right most marker, low reliability) to 9 (left most marker, high reliability). Labels for RI >= 2, 4 and, 6 are skipped for simplicity. For instance, 58% of all predictions in our cross-validation were made at reliability levels of 7 or higher (gray arrows). At this reliability, 95% of all effect predictions and 90% of all neutral predictions were correct.

The difference between the raw output units reasonably estimates prediction confidence [11,36]. We used this difference to define a reliability index (RI, Eqn. 6) and demonstrated its excellent correlation to prediction strength, *i.e*. the reliability index and performance (Figure 4). The final binary predictions (neutral/effect) of SNAP2 are calculated from the network outputs based on the user-defined decision threshold (default: -0.05). By moving the threshold, users can vary the accuracy-coverage balance. Higher thresholds result in more accurate predictions at the cost of covering fewer variants; lower thresholds cover more variants while reducing accuracy. By dialing through the entire threshold spectrum for our non-disease data (PMD/EC data), we estimated and fixed the default decision threshold (Figure 4A). To put this into perspective: when predicting effect/neutral for all variants, SNAP2 is correct in about 75% of its neutral predictions and in 86% of its effect predictions (Figure 4B rightmost points). If users focus on the 50% strongest predictions (Figure 4B; x-axis at 0.5), they could expect the ~92% of the neutral predictions and ~96% of the effect predictions to be correct (RI≥8, Figure 4B). Note that for the purposes of simplified visualization, to display SNAP2 reliability with one digit per residue (*e.g*. to view along with multiple sequence alignments), we projected the actual RI onto integers from 0 (low reliability - worst prediction) to 9 (high reliability - best prediction, Figure 4B).

## Discussion

### Performance related to experimentally biased balance of neutral vs. effect variants

Machine learning tends to work best when testing and training data are sampled from the same distribution. What are the true data that we want to assess our method upon? One proxy for this type of *truth *might be the next "one million variants" experiment: test 1,000 randomly selected naturally occurring variants in 1,000 representative proteins. One question is: how many variants will be identified as being neutral with respect to protein function? The answer remains importantly vague. Several seemingly contradictory findings are the following. On the one hand, for almost every sequence position (residue) there is a non-native variant that has very little effect on one particular experimental assay [56]. Loosely put: "sequence can change without effect". On the other hand, for almost every residue there is a variant that affects function somehow [56]. Loosely put: "every residue in a protein matters and its variation can change function". There is evidence that individuality of people is partially caused by many slightly non-neutral variants [19]. However, this does not help in estimating the "true" ratio neutral/effect for the next one million. Clearly, today's data sets are strongly biased toward effect variants, simply because it is simpler to measure and easier to publish an effect than a neutral variation. Unfortunately, most of our performance estimates crucially depend on the *true *ratio neutral/effect. Thus, our estimates remain almost as incomplete as the experimental data.

### What to expect from variant prediction?

Methods that identify variants related to disease try to pick up changes that are strong enough to cause phenotypic effects that can be classified as disease. This is difficult for two reasons. Firstly, the causality between variant and disease is only clear for the simplest cases such as monogenic or Mendelian diseases. Most diseases appear to be complex, in the sense that they are onset only in the presence of several variants and proper environmental conditions. GWAS have shown that variants associated with disease are found in healthy individuals, and vice versa. Loosely put: the definition of a disease variant may depend on other variants present in the particular genotype of the phenotype carrier. Secondly, even for seemingly clear-cut cases, the classification of "disease" might be misleading. Consider the example of the sickle-cell anemia variants of the hemoglobin B-chain, which can result in a number of chronic health problems on the one hand but grant immunity to some malaria types on the other. In other words, the definition of a disease variant may depend on the environment of the individual.

In contrast to disease, the prediction of the effect of a variant upon molecular function focuses only on the native function of one particular protein. For many examples, such effects are independent of the individual and, often (although not always), of the environment. However, such a focus bears another set of problems: (1) Today's computational methods cannot reliably distinguish between gain and loss of function. They simply predict whether or not the mutation affects native function at all. (2) It is often difficult to relate the strength of a functional effect to its biological relevance. For instance, a "bit" of change in p53 functionality may cause severe phenotypes, whereas a "large" functional effect on other proteins may have little biological impact. In other words, predicted effects have to be put into perspective of the protein in question.

### SNAP2 not limited to human variants

Functional effects of sequence variations are not limited to pathogenicity in humans. As most experimental data are human-centric, and as the disease variants are generally most consistent with functional effect [17], SNAP2 performed best for those. This might also explain why for these SNAP2 performed similar to PolyPhen-2 that has been optimized to human data. On non-human variants, however, SNAP2 predictions were most accurate and reliable as compared to other methods. This suggests SNAP2 as a valuable tool for the preliminary analysis of variants in any organism. Specifically, SNAP2 might be the ideal starting point for the comparison of variants between species, *e.g*. human vs. chimp vs. mouse.

### Neutral variants predicted worse

All methods performed significantly better for effect than for neutral variants. This in agreement with findings reported in Bromberg *et al *[19] and can be explained in two ways.

(1) The imbalance might originate from incomplete experimental evidence. The effect of variants is typically evaluated on the basis of one or a few phenotypes/assays. If these produce no visible difference as compared to wild-type control the variant is reported as neutral. However, it might still have an effect on other assays that are not performed.

(2) The variants for experimental analysis are usually not selected at random. Instead, researchers prudently focus on the most important changes; often those changes are related to diseases. Such a prioritized selection samples the feature space incompletely. This may hamper computational detection of relevant patterns for neutral variants. The incomplete sampling may also skew performance estimates: the variants most trivially expected to be neutral might be predicted by the methods but might not be tested experimentally because they are simple to guess. For this reason, comprehensive testing as performed for the *E. Coli *LacI repressor or the HIV-1 protease is an invaluable source of information for computational prediction of variant effects. Such data will likely be crucial in overcoming the neutrality dilemma and will significantly further our understanding of the underlying molecular mechanisms of variant effects.

### SNAP2_noali _succeeded where others failed

We specifically trained a classifier to predict functional effects without using evolutionary information. This unique novel resource might become increasingly useful as ongoing sequencing efforts bring in more data. The current release of the UniRef50 (March 2014) contains ~9.5 million sequence clusters of which over 6.5 million (~68%) contain only one protein, *i.e*. are proteins so far unique to one organism. For those over 6.5 million, very little evolutionary information is available to guide other variant effect predictions and the fraction of orphan clusters appears to be increasing; *i.e*. in October 2012, the UniRef50 contained ~64% orphan clusters - a 4% increase over 1.5 years. This difference might originate from the decreasing quality of increasing sequencing data. However, a similar trend had been observed 12 years ago with arguably more accurate sequencing data [57]. Except for SNAP2_noali_, all methods perform significantly worse for orphans and, in some cases, at the level of throwing a coin. Often they produce no results, which also is at the random level. By including a variety of specific features, we developed a classifier that still achieves a two-state accuracy Q2 around 68% from sequence alone even for these 6.5 million orphan families. This unique type of predicted information might become very relevant for uncharacterized protein families.

### Best prediction of difficult cases

By comparing predictions for variants for which commonly applied methods disagreed, we extracted variants that were difficult to classify. For these difficult cases, our new method SNAP2 significantly outperformed SNAP (set ALL-difficult: Q2(snap2) = 69%, Q2(snap) = 53%) and SIFT (Q2(sift) = 41%). For the difficult variants from human PMD, SNAP2 performed just as well as PolyPhen-2, although this comparison gave PolyPhen-2 an unfair advantage because the data set used had partially been used to train PolyPhen-2.

### More and better data needed to advance further?

SNAP2 and PolyPhen-2 reached similar levels of performance with rather different approaches, but we made so many so important changes to SNAP that we were surprised not to improve more. Was this because prediction performance has reached a plateau, *i.e*. have we reached the limits for a method using only sequence information as input? Many observations suggest that our data sets remain importantly incomplete. For instance, we observed that our EC data was inconsistent but that we fared worse by leaving it out. We improved a little through the addition of the OMIM data, but possibly only so much so because the data had implicitly already been predicted correctly [17]. In other words: OMIM samples exhibit, on average, extreme signals that are somewhat 'easy' to predict. Thus, adding samples from the top end of the effect distribution did not help improve our prediction of difficult cases where we often find unclear/contradicting signals. Another indication of incompleteness of experimental data was the result that we needed to use all available data to achieve peak performance, *i.e*. smaller subsets reduced performance (data not shown). Still, are we close to a saturation of performance, or can we expect another leap? The lessons learned from advancing secondary structure prediction through the combination of machine learning and evolutionary information suggest that there is yet no way to tell.

## Conclusions

We significantly improved over our seven-year-old method SNAP for the prediction of functional effects from single point variants or mutations in the amino acid sequence. SNAP2, the new method improved through more and better data and through more input features. SNAP2 annotates functional effects of variants with little preference to particular species and/or particular types of effects. This allows users to perform bias-free cross-species comparisons, such as looking at sequence positions that differ between human and mouse. We believe that this might be helpful for understanding and predicting disease-causing variation, as well as for facilitating drug development. A measure of prediction reliability (Reliability Index; RI) allows users to focus on the most promising candidates. Additionally, a big achievement of this work is the development of SNAP2_noali _- a model that predicts effects of variants without using evolutionary information. Ongoing deep-sequencing efforts bring in novel sequences and novel variants alike. Many of these variants occur in sequences without families. Possibly for millions of proteins SNAP2_noali _provides a reliable prediction of variant effects and allows for a quick assessment of functionally relevant positions in novel proteins. Both versions of SNAP2 have been optimized towards runtime efficiency to enable large-scale *in silico *mutagenesis studies that probe the landscape of protein mutability [56,58] to learn important news about protein structure and function.

## Competing interests

The authors declare that they have no competing interests.

## Authors' contributions

MH, YB, and BR conceived this work and designed the experiments. MH wrote the software and carried out the experiments. MH and YB collected the data and analyzed the results. MH, YB, and BR wrote, revised, and approved the manuscript.

## Supplementary Material

Additional file 1 Click here for file
