# Supplementary material for: Better prediction of functional effects for sequence variants
Source: BMC Genomics. 2015 Jun 18;16(Suppl 8):S1. doi: 10.1186/1471-2164-16-S8-S1 (PMC4480835; doi:10.1186/1471-2164-16-S8-S1)
Supplement: Additional file 1 [file 1471-2164-16-S8-S1-S1.pdf]

Supporting online material for: Better prediction of functional effects for sequence variants

Maximilian Hecht, Yana Bromberg & Burkhard Rost

Table of Contents for Supporting Online Material

1. Input feature calculation
2. Table SOM_1: Input features selected from AAindex
3. Table SOM_2: Performance on independent data sets
4. Table SOM_3: Performance estimates on ALL data set
5. Figure SOM_1: Accuracy-Coverage cruves on ALL data set
6. Figure SOM_2: Score distribution for SNAP2 on ALL data set

Short description of Supporting Online Material

This SOM contains a detailed description of features and their extraction for use in the neural network predictor. We also included three tables (1) listing the cluster representatives from the AAindex database, that were selected as helpful features in $SNAP2_{noali}$, (2) a performance comparison on independent protein-specific data, namely the HIV-1 protease and the *Escherichia Coli* LacI repressor and (3) a table showing performance values on our comprehensive ALL (main manuscript, methods section) data set. Moreover, this SOM includes a figure (Fig. SOM_1) showing the performance of SNAP2 and $SNAP2_{noali}$ in comparison to SIFT and random predictions.

Material

Input feature calculation. In order to use amino acid and protein properties in neural networks these have to be presented as normalized numerical values. The following section describes the exact calculation or extraction of these values.

Delta features. Where applicable, we calculated *delta features* that describe the change in certain features between the native amino acid and its variant. All *delta features* are encoded by two nodes per residue: one for the "severity" (absolute difference between wildtype and mutant value) the other for the "direction" ('1' if positive and '0' if negative) of change.

Biophysical properties. In addition to mass, volume, charge, hydrophobicity and the presence of C-beta branching amino acids (as already present in SNAP) we collected one representative for each cluster of correlated amino acid indices from the AAindex database [1]. These indices are matrices containing values for each amino acid (or pair of amino acids) that cover a variety of amino acid properties and features derived from these (Table SOM_1). We extracted the corresponding (already normalized) value for each residue in the window, resulting in w input values. Then we calculated the two-node delta feature. The first node was the absolute difference between the wildtype and the mutant value.

Binding residues. We used ISIS [2] to predict the protein-protein binding sites and DISIS [3] to predict the protein-DNA binding sites. We extracted both the binary prediction (binding/non-binding) and the raw prediction score for each residue in the window (21 * 2 = 42 input nodes).

Disordered regions. We used the META-Disorder predictor tool (MD; [4]) tool to calculate a three-node disorder feature for all residues in the window: We extracted the binary per-residue prediction (disordered/not-disordered) and the prediction reliability.

Proximity to N- and C-terminus. We calculated the proximity of the variant position to each terminus individually as the normalized number of residues between terminus and the position of interest (2*1 = 2 input nodes).

Contact potentials. We extracted normalized distance-dependent statistical potentials (for contacts within 5 Ångstrøms=0.5nm) [5]. For both native amino acid and variant, we extracted the potential as a 20-node feature. Additionally, we calculated the delta values for this feature (difference between native and variant) for their eight (four residues before and after) sequence neighbors (20*2 + 8*2 = 56 input nodes).

Co-evolving positions. We estimated the co-evolution of positions in a multiple sequence alignment following the approach from [6]. For each position in the multiple alignment we used the OMES [7] algorithm to calculate the correlation

with any other position. The OMES method compares the observed co-occurrence of amino acid X at position i and amino acid Y at position j to the expected co-occurrence at positions i and j. This pairwise comparison yielded a ranking of all positions based on their pairwise correlation to any other position. From these, we extracted a six-node feature indicating the rank and the score (i.e. the deviation from the expectation value) for the three positions most correlated with the mutation position (2*3 = 6 input nodes).

Residue annotation. In addition to SWISS-PROT annotations and SIFT predictions as already used in SNAP we considered residue annotation from Pfam [8] and PROSITE [9] to describe native and variant amino acids: (i) We determined whether the position was part of a PfamA domain. If so, we collected metrics of domain conservation and the posterior probability of native and variant belonging to that domain (4 input nodes). (ii) From PROSITE we extracted a binary single-node feature for all residues in the window indicating whether the specific residue is part of a PROSITE pattern (21 input nodes).

Low-complexity regions. We used the SEG [10] algorithm to mask protein regions with low-complexity. From this masking, we extracted a feature of 21 binary input nodes indicating whether a mutation is in or close to a low-complexity region.

Global features. We added global sequence information by calculating four features: The amino acid composition as the relative frequency of each amino acid (20 amino acids + 1 unknown = 21 input nodes); the sequence length feature encoding the protein length in 6 bins (0-60, 61-120, 121-180,181-240, 241-300, >300; 6 input nodes); the secondary structure composition and the solvent accessibility composition, each as a twelve-node binary feature using four bins (0-25%, 26%-50%, 51%-75%, 76%-100%) for each state: helix-strand-other or buried-intermediate-exposed (2 * 12 = 24 input nodes).

Table SOM_1: Input features selected from AAindex *

AAindex accession [1]	Description
VINM940103	Normalized flexibility parameters (B-values) for each residue surrounded by one rigid neighbour [11]
BLAM930101	Alpha helix propensity [12]
DAYM780201	Relative mutability [13]
QIAN880123	Weights for beta-sheet [14]
KLEP840101	Prediction of protein function from sequence properties; Discriminant analysis of a data base: Net charge [15]
SNEP660101	Relations between chemical structure and biological activity in peptides: Principal component I [16]
RICJ880113	Relative preference values of amino acids at C2 [17]
SIMK990101	Distance-dependent statistical potential (contacts within 0-5 Angstroms) [5]

* We listed the best-performing input features, i.e. amino acid indices that were selected by the feature selection procedure. Other indices from the corresponding clusters performed similarly. For each of these features both window-based and delta features were included into the final sequence-only network $SNAP2_{noali}$.

Table SOM_2: Performance on independent data sets *

Method	LacI repressor	HIV-1 protease
SIFT	72.2% ± 1.0	79.5% ± 3.2
SNAP	72.0% ± 1.0	78.3% ± 3.0
SNAP2	78.3% ± 0.9	74.1% ± 3.2

- Shown is the overall two-state accuracy (Q2 value; Method section) on 4041 LacI mutants and 336 HIV-1 protease mutants for SIFT, SNAP and SNAP2.

Table SOM_3: Performance estimates on ALL data set.

	Q2	F1 (neutral)	F1 (effect)	MCC	ROC AUC
SNAP2	83.5%	0.79	0.87	0.65	0.91
SNAP	80.1%	0.76	0.83	0.59	0.88
SIFT	77.4%	0.74	0.83	0.54	0.84
PolyPhen-2	80.8%	0.75	0.84	0.60	0.85

* Performance estimates were obtained from cross-validation for SNAP2. For all methods the default thresholds were applied. Estimates are based on all variants from our ALL data set (see methods section; Data).

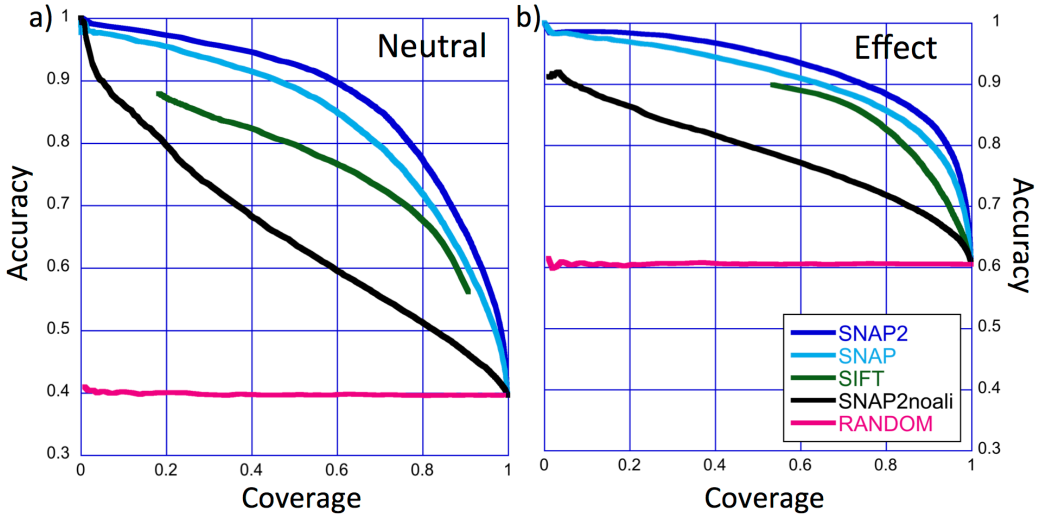

Figure SOM_1: Accuracy-Coverage curves for ALL data. These figures show performance on the *ALL* data set. Our new method SNAP2 (dark blue) outperforms its predecessor (SNAP, light blue), and SIFT (green) for both the variants that do not affect function (neutral, a) and for those that affect function (b). The x-axes indicate coverage/recall (Eqn. 1,2), *i.e.* the percentage of observed neutral (a) and effect (b) variants that are correctly predicted at the given threshold. The y-axes indicate accuracy/precision (Eqn. 1,2), *i.e.* the percentage of neutral (a) and effect (b) variants among all variants predicted in either class at the given threshold. The dark line ($SNAP2_{noali}$) marks the performance of a SNAP2 version that does not use any information from sequence alignment. All results are computed on the test sets not used in training. A pink line marks the performance of a random predictor.

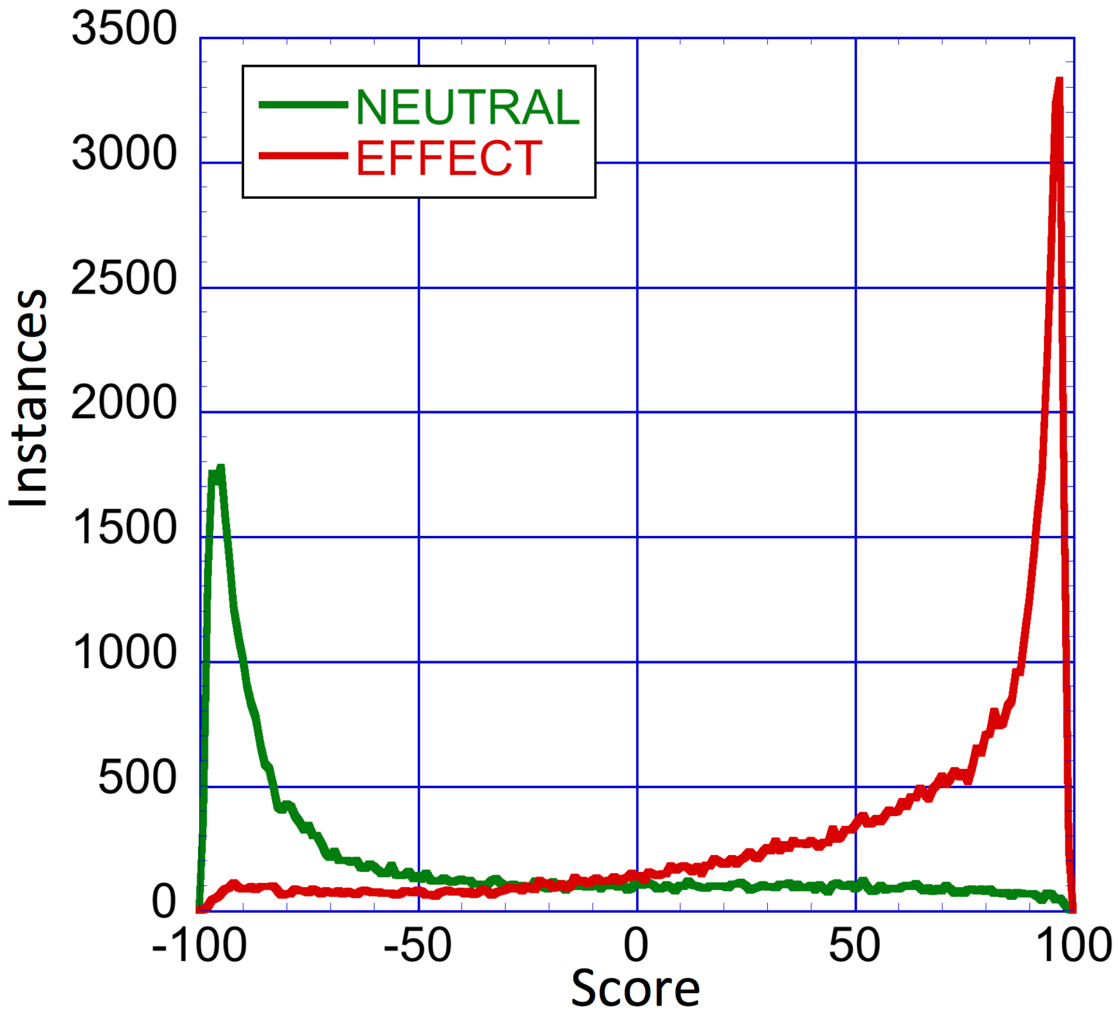

Figure SOM_2: Score distribution for SNAP2 on ALL data. Shown is the number of instance (y-axis) for each score (x-axis). Effect variants (red) mostly have predicted scores > 0 while neutral variants (green) are predominantly predicted at scores < 0.

References for Supporting Online Material

1. Kawashima, S. & Kanehisa, M. (2000). AAindex: amino acid index database. *Nucleic acids research* **28**, 374.

2. Ofran, Y. & Rost, B. (2007). ISIS: interaction sites identified from sequence. *Bioinformatics* **23**, e13-6.

3. Ofran, Y., Mysore, V. & Rost, B. (2007). Prediction of DNA-binding residues from sequence. *Bioinformatics* **23**, i347-53.

4. Schlessinger, A., Punta, M., Yachdav, G., Kajan, L. & Rost, B. (2009). Improved disorder prediction by combination of orthogonal approaches. *PloS one* **4**, e4433.

5. Simons, K. T., Ruczinski, I., Kooperberg, C., Fox, B. A., Bystroff, C. & Baker, D. (1999). Improved recognition of native-like protein structures using a combination of sequence-dependent and sequence-independent features of proteins. *Proteins* **34**, 82-95.

6. Kowarsch, A., Fuchs, A., Frishman, D. & Pagel, P. (2010). Correlated mutations: a hallmark of phenotypic amino acid substitutions. *PLoS computational biology* **6**.

7. Fodor, A. A. & Aldrich, R. W. (2004). Influence of conservation on calculations of amino acid covariance in multiple sequence alignments. *Proteins* **56**, 211-21.

8. Punta, M., Coggill, P. C., Eberhardt, R. Y., Mistry, J., Tate, J., Boursnell, C., Pang, N., Forslund, K., Ceric, G., Clements, J., Heger, A., Holm, L., Sonnhammer, E. L., Eddy, S. R., Bateman, A. & Finn, R. D. (2012). The Pfam protein families database. *Nucleic acids research* **40**, D290-301.

9. Sigrist, C. J., Cerutti, L., de Castro, E., Langendijk-Genevaux, P. S., Bulliard, V., Bairoch, A. & Hulo, N. (2010). PROSITE, a protein domain database for functional characterization and annotation. *Nucleic acids research* **38**, D161-6.

10. Wootton, J. C. & Federhen, S. (1993). Statistics of local complexity in amino acid sequences and sequence databases. *Computers & chemistry* **17**, 149-163.

11. Vihinen, M., Torkkila, E. & Riikonen, P. (1994). Accuracy of protein flexibility predictions. *Proteins* **19**, 141-9.

12. Blaber, M., Zhang, X. J. & Matthews, B. W. (1993). Structural basis of amino acid alpha helix propensity. *Science* **260**, 1637-40.

13. Dayhoff, M. O., Schwartz, R. M. & Orcutt, B. C. (1978). A model of evolutionary change in proteins. In *Atlas of protein sequence and structure* (Dayhoff, M. O., ed.), Vol. 5. National Biomedical Research Foundation, Washington, DC.

14. Qian, N. & Sejnowski, T. J. (1988). Predicting the secondary structure of globular proteins using neural network models. *Journal of molecular biology* **202**, 865-884.

15. Klein, P., Kanehisa, M. & DeLisi, C. (1984). Prediction of protein function from sequence properties: Discriminant analysis of a data base. *Biochimica et Biophysica Acta (BBA)-Protein Structure and Molecular Enzymology* **787**, 221-226.

16. Sneath, P. (1966). Relations between chemical structure and biological activity in peptides. *Journal of theoretical biology* **12**, 157-195.

17. Richardson, J. S. & Richardson, D. C. (1988). Amino acid preferences for specific locations at the ends of alpha helices. *Science (New York, NY)* **240**, 1648.
